# Increasing the Understanding of Nutrient Transport Capacity of the Ovine Placentome

**DOI:** 10.3390/ani14091294

**Published:** 2024-04-25

**Authors:** Cathrine Erichsen, Axel Heiser, Neville Haack, Paul Maclean, Cathy Mary Dwyer, Sue McCoard

**Affiliations:** 1 AgResearch Ltd., Grasslands Research Centre, Private Bag 11008, Palmerston North 4474, New Zealandaxel.heiser@agresearch.co.nz (A.H.);; 2 Scotland’s Rural College (SRUC), Easter Bush Campus, Edinburgh EH25 9RG, UK; cathy.dwyer@sruc.ac.uk; 3 Royal (Dick) School of Veterinary Studies, University of Edinburgh, Easter Bush Campus, Midlothian EH25 9RG, UK

**Keywords:** ovine, placenta, placentome morphology, amino acids, arginine, gene expression

## Abstract

**Simple Summary:**

The ovine placenta facilitates transport of oxygen and nutrients to the growing fetus. Placental morphology and placental function are affected by environmental factors, and the changes are not well understood. This study aimed to identify the effect of tissue type (caruncle vs. cotyledon), placentome morphological subtype, and maternal parenteral arginine supplementation in mid–late pregnancy on the placental nutrient transport capacity at 140 days of gestation, using a gene expression approach. Results highlighted that placentome morphology and tissue type are associated with differential expression of specific amino acid (AA) transporter genes, suggesting a potential adaptive response to increase the transport capacity of the placenta. Maternal arginine supplementation influenced expression of genes involved in AA transport, placental efficiency, and angiogenesis, which may affect the placental transport capacity. The results contribute to an increased understanding of placental function and provide new insights into the effects influencing the placental nutrient transport capacity to support fetal growth in sheep.

**Abstract:**

Placental nutrient transport capacity influences fetal growth and development; however, it is affected by environmental factors, which are poorly understood. The objective of this study was to understand the impact of the ovine placentome morphological subtype, tissue type, and maternal parenteral supplementation of arginine mono-hydrochloride (Arg) on nutrient transport capacity using a gene expression approach. Placentomal tissues of types A, B, and C morphologic placentome subtypes were derived from 20 twin-bearing ewes, which were infused thrice daily with Arg (*n* = 9) or saline (Ctrl, *n* = 11) from 100 to 140 days of gestation. Samples were collected at day 140 of gestation. Expression of 31 genes involved in placental nutrient transport and function was investigated. Differential expression of specific amino acid transporter genes was found in the subtypes, suggesting a potential adaptive response to increase the transport capacity. Placentomal tissues differed in gene expression, highlighting differential transport capacity. Supplementation with Arg was associated with differential expressions of genes involved in amino acid transport and angiogenesis, suggesting a greater nutrient transport capacity. Collectively, these results indicate that the morphological subtype, tissue type, and maternal Arg supplementation can influence placental gene expression, which may be an adaptive response to alter the transport capacity to support fetal growth in sheep.

## 1. Introduction

The placenta is the maternal–fetal interface and is a key regulator of fetal growth and development [1]. To enable this, nutrients and oxygen have to be supplied by the maternal blood stream. The function of the placenta is to facilitate passive and active nutrient exchange between the dam and fetus [2,3,4]. In ruminants, the placenta forms complexes called placentomes, which consist of the fetal cotyledonary and maternal caruncular components [5]. In sheep, the placentomes reach their full size at days 70 to 85 of gestation [6]. Thereafter, the vascularity, transport capacity, and morphologic structure of placentomes can alter in response to environmental signals (e.g., nutrient supply, hypoxia, and stress) to support conceptus growth with advancing gestation [7]. Numerous factors have been identified that affect placental function [8], including hormones [9,10], nutrition [11], and number of fetuses [12]. However, the mechanisms by which environmental factors influence placental function are poorly understood.

Vatnick et al. [13] were the first to identify that the ovine placentome changes morphology between four distinct subtypes, from A to D, where the hemophagous zone becomes more emerged from A to D placentomes, and their distribution becomes obvious in the last third of pregnancy. This has been hypothesized to be a reaction to stress factors, such as hypoxia, nutrient restriction, and/or multiple fetuses [7]. This change may be an adaptation to generate a more efficient placenta to increase nutrient supply to the growing fetus(es) [7,11,12], potentially via increased placental vascularity, which subsequently increases the nutrient transport capacity [14]. We have recently reported that birth rank (single vs. twin) and placentome subtype influence placental gene expression, notably, changes in amino acid (AA) transport and metabolism, oxidative stress, and angiogenesis and/or blood flow [15]. These results indicate that both maternal and fetal factors may influence placental function in sheep and that alterations in gene expression and/or placentome morphological shifts may contribute to adaptations to support fetal growth in twin pregnancies.

Arginine is a conditionally essential AA, meaning that, although it can be synthesized by the body, it must be supplemented through diet when rates of utilization are greater than rates of synthesis [16]. Among others factors, Arg plays a key role in multiple placental physiological pathways important for blood flow, the antioxidant defense system, and protein synthesis [17], and parenteral Arg supplementation of twin-bearing ewes from 100 to 140 days of gestation is associated with a change in placentome morphology [18]. In that study, placentas of Arg-supplemented ewes had greater proportions of everted type B placentomes than type A compared to un-supplemented control ewes, which had equal proportions of A and B subtypes. Arg supplementation of ewes in that study was also associated with positive effects on brown adipose tissue mass and function in both sexes and on birth weight of female lambs [19,20]. These observations suggest that maternal Arg supplementation may influence placentome morphology, with subsequent effects on nutrient transport capacity to support fetal growth and development. Therefore, this provides a model with which to evaluate the effect of placentome subtype as well as Arg supplementation on the placental nutrient transport capacity.

The objective of this study was to understand the impact of placentome morphology and maternal parenteral Arg supplementation on placental function (notably, transport capacity) using a gene expression approach. The hypothesis tested was that maternal Arg supplementation and eversion of placentome subtypes would be associated with changes in the expression of genes involved in placental nutrient transport and/or angiogenesis.

## 2. Materials and Methods

### 2.1. Experimental Design

The placental samples used were collected from a prior study that investigated the effects of parenteral Arg supplementation to twin-bearing ewes on placental development and the growth and development of the offspring. The experimental design and methods for the animal study have been previously described [18,20] and were approved by the University of Auckland Ethics Committee (C889). Briefly, placental tissue was collected from 20 twin-bearing Romney ewes naturally mated to Poll Dorset sires. From day 100 of gestation to day 140, the ewes received either a parenteral bolus of Arg-mono-hydrochloride (L-Arg-HCL; Merck KGaA, Darmstadt, Germany; 345 µmol/kg BW; *n =* 9) or approximately the same volume of sterile saline (*n =* 11), three times daily. At day 140 of gestation, the ewes were euthanized, the gravid uterus was opened along the anti-mesometrial side, and the fetuses were removed. Individual placentomes from each individual placenta (one per fetus) were dissected and categorized into morphologic types A to D based on the classification system developed by Vatnick et al. [13], as described previously [18]. The classification was performed by the same investigator to reduce between-operator variability. Subsequently, the placentomes were manually separated into their caruncular and cotyledonary components and individually weighed. Cotyledon and caruncle tissue from each placentome type were immediately snap-frozen in liquid nitrogen at –196 degrees Celsius. Only cotyledon and caruncle tissue from type A, B, and C heavy placentomes (>4.1 g) were used in the present study, as these were the most represented and there were insufficient type D placentomes to enable statistical comparison.

### 2.2. RNA Extraction

RNA was isolated from 108 cotyledon and caruncle tissues from type A, B, and C heavy placentomes from placentas supporting 18 male and 22 female fetuses from Arg and Ctrl ewes (Table 1). Heavy placentomes were selected for this study, as these were the most abundant. RNA was prepared from both caruncular and cotyledonary tissue using spin columns (RNeasy Mini, Qiagen, Hilden, Germany) following the manufacturer’s recommendations. The method for RNA extraction has previously been described by McCoard et al. [15]. Briefly, approximately 25–30 mg of frozen placental tissue was used for each sample. For the caruncular placental tissue, disruption of the frozen tissue was performed using a mortar and pestle under liquid nitrogen, followed by homogenization in 600 µL of buffer RLT (Qiagen, Hilden, Germany) using a needle (20 G) and syringe. Homogenates were centrifuged, 300–400 µL of supernatant was taken, and one volume of 70% ethanol was added. A 300 to 400 µL mixture of supernatant and ethanol was then added to the spin column. For the cotyledonary tissues, the samples were thawed and homogenized using a mixture of zirconium beads, consisting of 0.5 g of 1 mm beads (Biospec Products Bartlesville, OK, USA) and seven to eight 3 mm beads (Benchmark Scientific, Sayreville, NJ, USA). Homogenization was performed using Buffer RLT using a TissueLyser II homogenizer (Qiagen, Auckland, NZ) at 30 Hz for 5 min. The solid debris was pelleted by centrifugation, clear lysed, and then added to the spin columns. RNA was eluted from the columns in two steps using 50 µL of RNase-free water each. Quantity and quality were assessed using a Nanodrop spectrophotometer (Thermo Fisher Scientific Inc, Waltham, MA, USA) with the minimum criteria of >30 ng of RNA available per reaction with an A260/A280 ratio of >1.7. Thereafter, the RNA was frozen at −80 °C until nano-string analysis.

### 2.3. Gene Expression Profile

A custom-designed code set (Nanostring Technologies, Seattle, WA, USA) was developed for 31 target genes and 3 reference genes. These genes were selected based on previous knowledge of their importance for placental nutrient transfer and fetal growth and development, particularly: (1) their importance for AA and glucose transport in the ovine placentome, (2) their relevance for optimal placental function, focused on vasculature, growth factors, hypoxia, and cortisol indicators, and (3) their involvement in arginine transport and metabolism in the placenta (Table 2).

Gene expression analysis was performed using the nCounter Analysis System (Nanostring Technologies, Seattle, WA, USA), as previously described by McCoard et al. [15]. The nano-string methodology was chosen, as it enables the direct measurement of mRNA expression levels of multiple genes from one sample with an equivalent sensitivity to real-time PCR [21,22]. The caruncular samples were prepared using the nCounter XT CodeSets system according to the manufacturer’s instructions (nCounter^®^ Gene Expression Assay User Manual, Nanostring, Seattle, WA, USA). Briefly, the RNA samples were thawed on ice and adjusted to 300 ng of RNA per reaction, either by dilution with DEPC-treated water or by concentration using a DNA130 SpeedVac Concentration System (Thermo Fisher Scientific Inc, Waltham, MA, USA). For the cotyledonary tissue, the samples were prepared using the ‘nCounter PlexSet Reagents for Gene Expression User Manual’ (Nanostring, Seattle, WA, USA).

### 2.4. Data Processing

The raw data were retrieved as reporter code count (RCC) files and imported into nSolver Analysis Software 4.0 (Nanostring, Seattle, WA, USA), where they underwent sample quality control according to the manufacturer’s recommendations (nCounter Gene Expression Data Analysis Guide, MAN-C0011-04, Nanostring, Seattle, WA, USA). The data processing methods have previously been described by McCoard et al. [15].

### 2.5. Statistical Analyses

Prior to statistical analysis, gene expression counts were log-transformed to achieve normal distribution of residuals. Assumptions of normality and equal variance were checked using residual plots. For each gene, mixed-effect models were performed on the log-transformed counts in R version 4.0.2 (R Core Team (2020). R: A language and environment for statistical computing; R Foundation for Statistical Computing, Vienna, Austria; https://www.R-project.org/, accessed on 22 June 2020) using the “lmer” function from the lmer4 package version 1.1.-26 [23] and fitting linear mixed-effects models using “lme4” (arXiv preprint arXiv:1406.5823). The model included tissue type (caruncle versus cotyledon), placentome morphologic type (type A, B, and C), supplementation (Arg and Ctrl), and sex (male versus female) as fixed effects, and ewe as a random effect. Two-way interactions were also investigated. No sex effects were observed but it was kept in the model as a fixed effect. The models were evaluated using the “anova” function from the lmer Test R package version 3.1-3 [24], and the “predictmeans” package version 1.0.4 [25] was used to produce back-transformed modeled means, confidence intervals, and pairwise *p*-values. *p*-Values of ≤0.05 were considered statistically significant.

## 3. Results

The main focus of this study was to investigate the effect of placentome subtype, tissue type, and maternal Arg supplementation on placental nutrient transport. Results of the main effects for supplementation, subtype, and tissue type are presented.

### 3.1. Abundance of Genes in the Placenta

**Figure 1:**
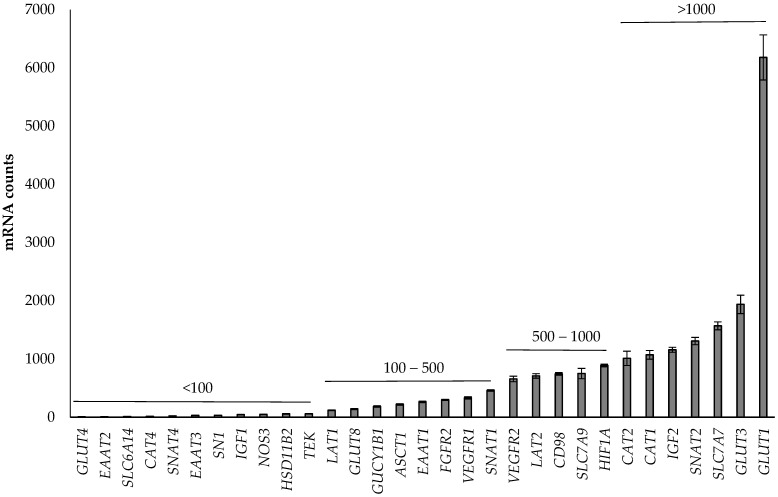
Abundance of the 31 genes in the ovine placentas (caruncle and cotyledon data) investigated, categorized by mRNA counts below 100, between 100 and 500, between 500 and 1000, and above 1000.

Overall, gene expression of the 31 genes evaluated varied considerably, with *GLUT1* being the most abundant and *GLUT4* the least abundant in the placentas (Figure 1).

### 3.2. Tissue Effects

**Figure 2:**
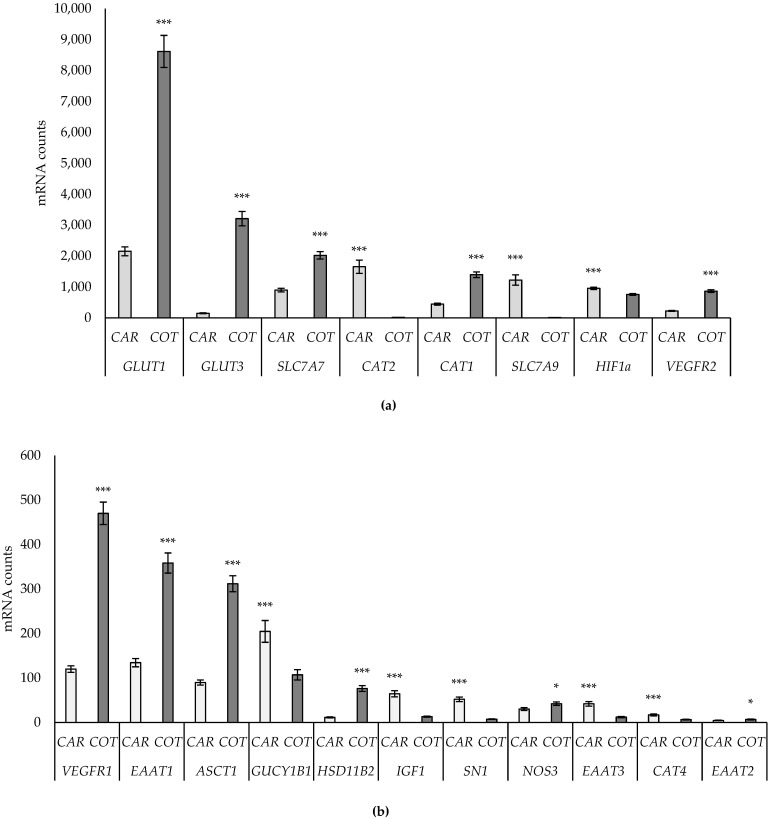
Effect of ovine tissue type (caruncle versus cotyledon) on expression of genes with mRNA counts with high (**a**) and low abundance (**b**). Data are presented as mean mRNA counts ± SE. Asterisks indicate differences between groups (* *p* < 0.05 and *** *p* < 0.001).

The main tissue (caruncle vs. cotyledon) effects are presented in Figure 2. Tissue differences were found for 19 of the 31 genes investigated: *GLUT1, GLUT3, SLC7A7, SNAT2, CAT1, VEGFR2, VEGFR1, EAAT1, ASCT1, HSD11B2, NOS3*, and *EAAT2* had higher expression in cotyledon than caruncle tissue, whereas *CAT2, SLC7A9, HIF1A, GUCY1B1, IGF1, SN1, EAAT3*, and *CAT4* had higher expression in caruncle than cotyledon tissue. No differences were found for the remaining genes.

### 3.3. Effects of Placentome Subtype

**Figure 3:**
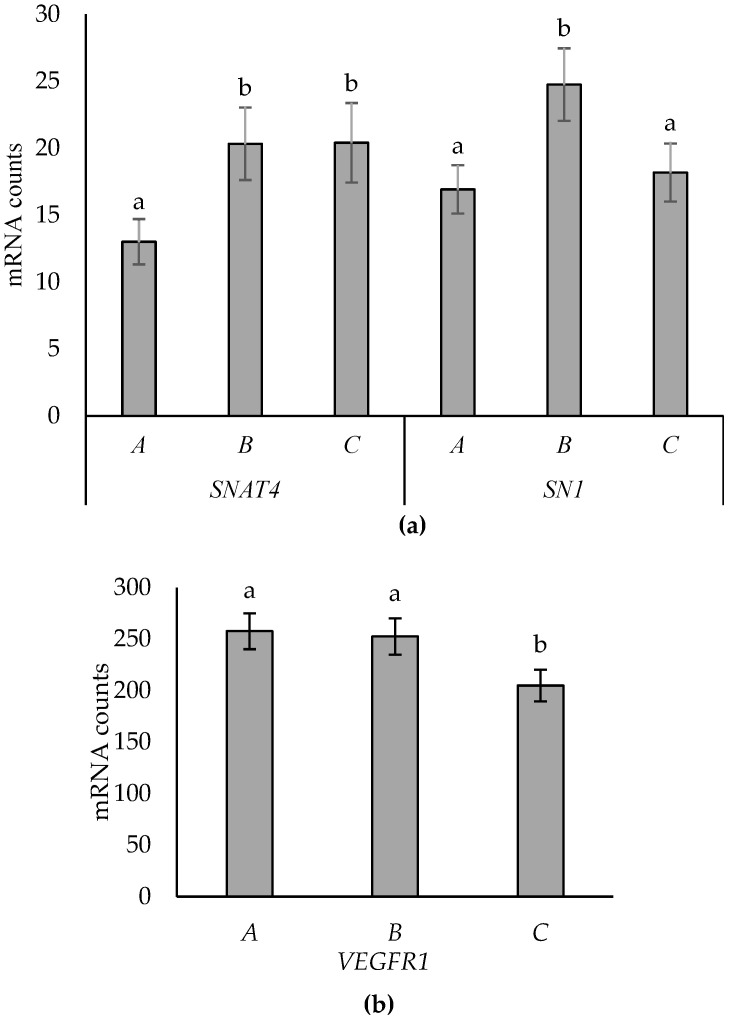
Effects of placentome subtype (A versus B versus C) on the expression of *SNAT4* (*p* = 0.015; **a**), *SN1* (*p* = 0.028; (**a**)), and *VEGFR1* (*p* = 0.049; (**b**)). Data are presented as mean mRNA counts ± SE. Superscripts with different letters indicate differences between subtypes.

Expression of *SNAT4, SN1*, and *VEGFR1* in the placentome subtypes is shown in Figure 3. Expression of *SNAT4* was lower in type A placentomes than types B and C, which did not differ. *SN1* expression was higher in type B compared to A and C, which did not differ. Expression of *VEGFR1* was higher in types A and B compared to C. No interactions with supplementation, tissue, or sex were evident for these genes.

**Figure 4:**
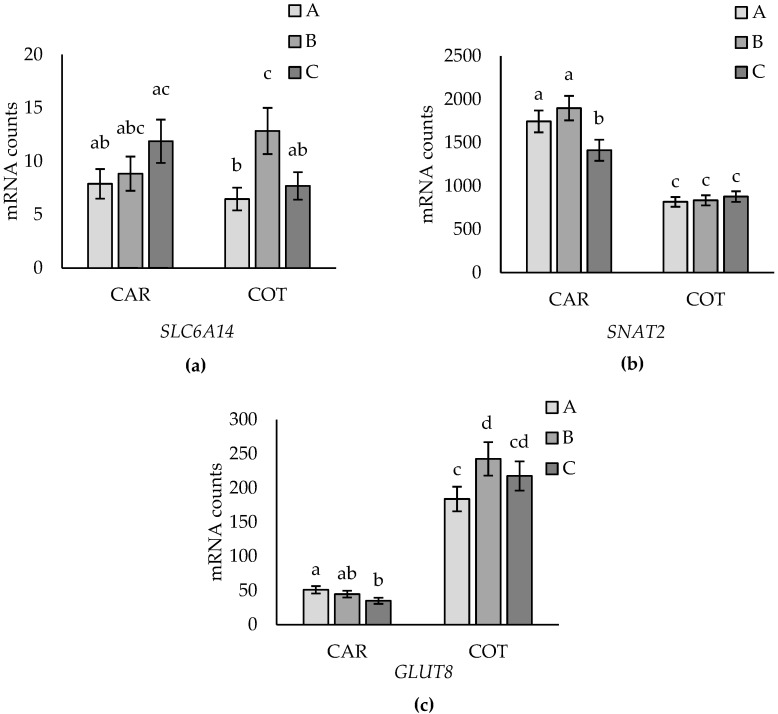
Placentome subtype (A versus B versus C) by tissue (caruncle (CAR) versus cotyledon (COT)) interaction for *SLC6A14* (*p* = 0.045) (**a**), *SNAT2* (*p* = 0.022) (**b**), and *GLUT8* (*p* = 0.026) (**c**). Data are presented as mean mRNA counts ± SE. Superscripts with different letters indicate differences between subtypes and tissues.

Two-way interactions between placentome subtypes (A–C) and tissue (caruncle vs. cotyledon) for *SLC6A14*, *SNAT2*, and *GLUT8* are shown in Figure 4. Expression of *SLC6A14* did not differ between placentome subtypes in caruncles, while in cotyledons, expression was higher in type B than in A and C, which did not differ (Figure 4a). *SNAT2* was more highly expressed in caruncles from types A and B compared to type C placentomes but did not differ between subtypes in cotyledon tissues (Figure 4b). Expression of *SNAT2* was also higher in caruncles than cotyledon tissues. *GLUT8* expression was higher in cotyledon than caruncle tissue. In caruncles, *GLUT8* expression was higher in type A compared to type C placentomes, while type B was intermediate and not different to the A and C subtypes. In contrast, *GLUT8* expression in cotyledons was higher in type B than type A, while type C placentomes were intermediate (Figure 4c). No other main effects or two-way interactions were observed for any of the genes evaluated.

### 3.4. Supplementation Effects

**Figure 5:**
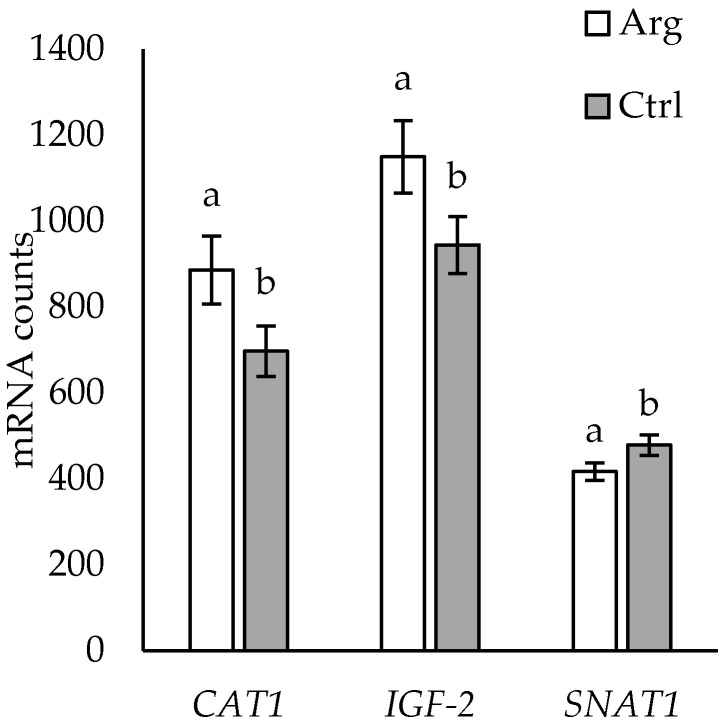
Overall supplementation (arginine (Arg) versus control (Ctrl)) effects for *CAT1* (*p* = 0.044), *SNAT1* (*p* = 0.030), and *IGF2* (*p* = 0.046) in placentomes of twin-bearing ewes. Data are presented as mean mRNA counts ± SE. Superscripts with different letters indicate differences between Arg and Ctrl placentome treatments.

Expressions of *CAT1* and *IGF2* were higher, while expression of *SNAT1* was lower in placentomes of Arg-supplemented than in Ctrl ewes (Figure 5).

**Figure 6:**
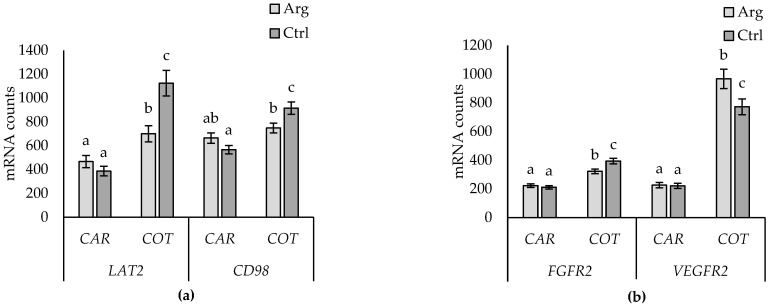
Tissue (caruncle (CAR) versus cotyledon (COT)) and supplementation (arginine (Arg) versus control (Ctrl)) interaction for *LAT2* (*p* < 0.001; (**a**)) and *CD98* (*p* = 0.003; (**a**)), and *FGFR2* (*p* = 0.020; (**b**)) and *VEGFR2* (*p* = 0.001; (**b**)). Data are presented as mean mRNA counts ± SD. Superscripts with different letters indicate differences between Arg and Ctrl treatments.

A two-way interaction between supplementation and tissue (caruncle vs. cotyledon) was found for *LAT2* and *CD98* (Figure 6a), whereby expression of both genes was lower in cotyledon tissue of Arg-supplemented than that of Ctrl ewes but did not differ in caruncle tissue. A two-way interaction between supplementation and tissue (caruncle vs. cotyledon) was also found for *FGFR2* and *VEGFR2*, which were unaffected by supplementation in caruncles, while *FGFR2* expression was lower, and *VEGFR2* higher, in cotyledon tissue from Arg-supplemented compared to Ctrl ewes (Figure 6b). No other main or two-way effects of supplementation were found for the other genes evaluated.

### 3.5. Supplementation-by-Subtype Interactions

**Figure 7:**
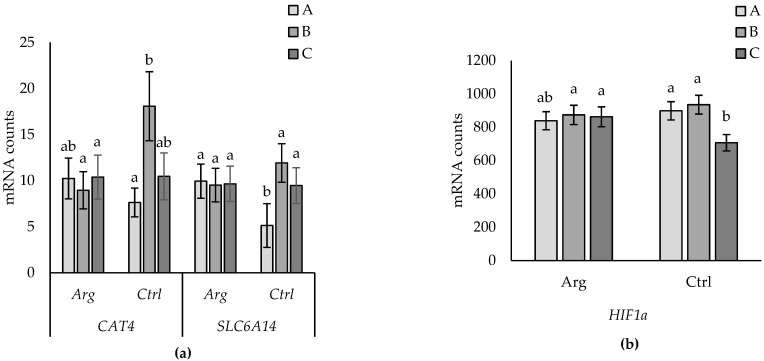
Subtype (A versus B versus C) by supplementation effect for *CAT4* (*p* = 0.043) and *SLC6A14* (*p* = 0.021) (**a**), and subtype-by-supplementation effect for *HIF1a* (*p* = 0.030) (**b**). Data are presented as mean mRNA counts ± SD. Superscripts with different letters indicate differences between Arg and Ctrl treatments.

Supplementation-by-subtype interactions for *CAT4* and *SLC6A14* are shown in Figure 7. *CAT4* expression did not differ between subtypes in placentomes from Arg-supplemented ewes, but expression was higher in type B than type A but not in type C placentomes from Ctrl ewes. Expression of *SLC6A14* did not differ between subtypes from Arg-supplemented ewes but was lower in type A than in B and C subtypes in placentomes from Ctrl ewes. A subtype-by-supplementation interaction was observed for *HIF1a*, where expression did not differ between placentome types in Arg-supplemented ewes, but in Ctrl ewes, expression was lower in subtype C compared to types A and B (Figure 7b). No other effects were found for the other genes evaluated.

## 4. Discussion

This study investigated the effects of placentome morphology and maternal Arg supplementation on gene expression of nutrient transporters and factors involved in the regulation of placental nutrient transport capacity and/or function. Investigation of gene expression in placental tissue was able to identify distinct differences in the abundance of genes in the placenta and in the cotyledon and caruncle. The key findings of this study were that type B placentomes generally showed increased gene expression of AA and glucose transporters compared to subtypes A and C, which partly supports the hypothesis that the morphologic shift from A to B increases transport capacity. Maternal parenteral supplementation with Arg was also associated with changes in the expression of genes involved in AA transport and angiogenesis, which suggests greater nutrient transport capacity in Arg-supplemented placentas compared to Ctrl, potentially in a morphologic-subtype-specific manner.

### 4.1. Abundance of Genes in the Placenta

The present study demonstrated *GLUT1* to be the most abundant gene of the 31 genes investigated. During late pregnancy, placental glucose requirements increase dramatically, as the fetus’ glucose requirements increase with the increase in fetal growth as well as the growing metabolic needs of the placenta [26,27]. Molina et al. [26] demonstrated that uteroplacental tissues consumed more than 80% of the glucose taken up by the uterus at days 71–81 of gestation. Furthermore, Currie et al. [27] found a 10-fold increase in the net transfer rate of glucose over the second half of pregnancy in ewes examined at mid-gestation (approximately day 76) and late gestation (approximately day 131). Glucose transport is predominately facilitated by *GLUT1*, followed by *GLUT3* [28,29]. Consistent with these observations, this study found *GLUT1* followed by *GLUT3* to be the most abundant genes in the placenta, consistent with our prior study [15], highlighting the major demand for glucose and the role of the placenta in regulating glucose supply to the fetus.

*GLUT8* and *GLUT4* have previously been described in the ovine placenta [15,30,31], but their importance may be minor [32]. Consistent with prior studies [15,30,31], the abundance of GLUT8 and GLUT4 was low in ovine placental tissues in the present study.

Of the AA transporter genes investigated, *SLC7A7* was the most abundant in the placenta, followed by *SNAT2*. *SLC7A7* has a high affinity for cationic and neutral AAs and has previously been identified in the ovine placentome and influenced by nutrient availability [32,33]. The present study highlights that *SLC7A7* may be a predominant AA transporter in the ovine placenta, which is consistent with our prior study [15]. *SNAT2* is an isoform of the AA transporter system A and has previously been described to transport neutral AA in mammalian placentas [8]. In mice, rats, and humans, *SNAT2* is postulated to be involved in placental efficiency, as more efficient placentas commonly tend to have higher *SNAT2* expression [34]. However, we have previously reported a negative correlation between *SNAT2* expression and total cotyledon weight in twin placentae [15], suggesting potential species-specific differences. The present study and our prior study [15] both highlight that SNAT2 is highly expressed in the ovine placenta, suggesting a potentially important role in placental AA transport in sheep.

### 4.2. Tissue Differences

Distinct differences in gene expression were identified between caruncles and cotyledons in this study. The results showed higher expression of some genes in cotyledons than caruncles, suggesting that placental–fetal transport of glucose and certain AAs is greater than maternal–placental transport. The most distinct differences to highlight are the higher expression of the glucose transporters *GLUT1* and *GLUT3* and system y+ AA transporter system genes *CAT1* and *CAT2* in cotyledons than in caruncles. Furthermore, receptors for VEGF, *VEGFR1*, and *VEGFR2* were more highly expressed in the cotyledons than caruncles. These results are in accordance with those of Vonnahme et al. [35], where higher levels of *VEGFR1* and *VEGFR2* were identified in cotyledons compared to caruncles of placentas from single-, twin-, and triplet-bearing ewes at day 140 of gestation. In accordance with Vonnahme et al. [35], it may be that vascular permeability to promote nutrient transport capacity is higher in cotyledons than caruncles in late pregnancy.

In summary, the results highlighted distinct physiological differences between tissue types, which should be taken into consideration in future studies.

### 4.3. Placentome Morphology

Changes in the placentome morphologic subtype (shift from A to D) have previously been postulated to be an adaptation to improve nutrient supply/change to the fetus [11,15]. In this study, four AA transporter genes (*SNAT4, SN1, SLC6A14,* and *SNAT2*), one angiogenic marker (*VEGFR1*), and one glucose transporter (*GLUT8*) were differentially expressed between placentome subtypes, while no differences in the other AA transporter genes, angiogenic markers, and glucose transporters, nor the hypoxia, growth factors, or stress markers, were evident. Insights into the potential roles of each of these genes in supporting placental nutrient transport/function are discussed.

Amino acids play a key role in fetal growth and development as well as in placental function [34]. In this study, the expression of 17 genes representing the AA transporter systems A, l, y+, y+L, ASC, N, b, B, and X were investigated, which have been classified based on their preference for neutral, acidic, or basic AA [36]. Placentome subtype effects were found for transporter systems A (*SNAT2, SNAT4*), N (*SN1*), and B (*SLC6A14*). An AA common to each of these gene transporters is glutamine [31,37,38,39,40,41]. Glutamine is transported to the fetus at a higher rate than any other AA at late gestation [41,42]. Glutamine is part of the glutamate–glutamine cycle between the placenta and fetal liver [43]. Glutamate is produced by the fetus and transported to the placenta, where it is synthesized to glutamine [41,42]. Glutamine is then transported back to the fetal blood stream, where it acts as an energy supply, protein building block, and signal for cell proliferation [1,41,43,44]. Therefore, transport of glutamine is highly prioritized in the placenta to support fetal growth and development [36]. Thus, the greater expression of *SNAT2* in type A and B placentomes, greater expression of *SNAT4* in type B and C placentomes, *SN1* expression in type B, and expression of *SLC6A14* in type B cotyledons, compared to the other subtypes evaluated, could indicate differences in transport capacity between morphologic subtypes with a preference for type B placentome subtypes.

The most abundant glucose transporter was *GLUT1*, followed by *GLUT3*, *GLUT8*, and *GLUT4*; however, only *GLUT8* expression was differentially expressed between placentome subtypes. *GLUT8* was first described in the ovine placenta by Limesand et al. [45], whereby *GLUT8* mRNA concentrations were found to increase during late gestation from day 90 to 135. Furthermore, Limesand et al. [45] found that fetal hypoglycemia resulted in decreased *GLUT8* mRNA concentrations, suggesting that decreased glucose transport could be influenced by decreased *GLUT8* expression. The greater expression of *GLUT8* in type B cotyledons compared to types A and C suggests that the morphological shift from type A to type B may be associated with an increase in the capacity to transport glucose. Lower expression of GLUT8 in type A compared to type B cotyledons was consistent with our prior observations [15]. Further, the tissue-specific differences observed (i.e., differential expression in cotyledons but not caruncles), coupled with ~four times greater expression of *GLUT8* for all placentome subtypes in cotyledons compared to caruncles, suggests that cotyledonary expression of *GLUT8* may play an important role in placental adaptation to supply glucose to support fetal growth. Expression of *GLUT1, GLUT3*, and *GLUT4* was not affected by ovine placentome morphology, consistent with our prior study [15], suggesting they may not be involved in an adaptation response.

The expression of angiogenic factors between ovine placentome morphologic subtypes have previously been described by Vonnahme et al. [46], where no differences between placentome subtypes were observed, suggesting that morphology is not affected by factors involved in vascularity and angiogenesis. However, we have previously reported differences in gene expression for *GUCY1B3* and *TEK* between placentome morphologic subtypes, suggesting increased blood flow in type B compared to type C placentomes [15]. In the present study, six angiogenic and vascularity factors common to the studies by Vonnahme et al. [46] and McCoard et al. [15] were investigated (*VEGFR1* and *2, FGFR2, TEK, NOS3*, and *GUCY1B3*). No difference in expression of *VEGFR2, FGFR2, TEK, NOS3*, and *GUCY1B3* between placentome subtypes was observed, which is consistent with the results of Vonnahme et al. [46] but not those of McCoard et al. [15]. In contrast, *VEGFR1* expression was greater in subtypes A and B compared to C in the present study, which contrasts with prior studies [15,46]. *VEGFR1* is one of two high-affinity receptors for the vascular endothelial growth factor (VEGF) [47]. While binding of VEGF to *VEGFR2* induces angiogenesis through endothelial cell proliferation, migration, and vasodilation, the binding with *VEGFR1* may inhibit *VEGFR2*-mediated effects [47,48]. Therefore, greater expression of VEGFR1 in type A and B compared to type C placentomes suggests that vascularity may be lower in subtypes A and B, compared to type C. Potential reasons for the inconsistent results for *VEGFR1* between the present study and that of Vonnahme et al. [46] could be that all ewes in the present study were twin-bearing, while Vonnahme et al. [46] investigated placentas of 38 pregnant ewes of unknown litter size. Vonnahme et al. [46] retrieved sets of placentomes per ewe, while this study was able to collect from individual fetuses and, thus, had a greater sample size and thus statistical power. Additionally, Vonnahme et al. [46] used quantitative real-time PCR (qPCR) to identify angiogenic factors, while this study used nano-string. The benefits of nano-string are that the system can quantify gene expression of many genes in a single reaction with very low concentrations of RNA [21,49]. Nano-string can also detect gene expression levels directly without enzymatic reactions, via two sequence-specific probes. Therefore, the gene expression method used in this study (i.e., nano-string compared to qPCR) may be a more sensitive method to detect differences in gene expression. Despite using the same methodologies, the results of the present study also contrast with those of our prior study [15]. Potential reasons for the differences are unclear but may be due to breed-specific effects. It is unclear if the morphologic shift from type B to C is associated with beneficial effects for transport capacity. Further, as with our prior study [15], it was not possible to study gene expression patterns in type D placentomes in the present study to see distinct expression differences in the morphologic shift associated with altered fetal growth. Further investigation is needed to understand angiogenetic differences between placentome morphologic subtypes.

### 4.4. Arginine Supplementation

Supplementation with Arg to pregnant ewes has previously been described to result in a greater supply of Arg to the placenta [18,50], with effects on birth weight, brown adipose tissue, and behavioral effects in the lambs [19,20]. This study found effects of maternal Arg supplementation on expression of the AA transporter genes *SNAT1* (system A)*, CAT1* (system y+), *LAT2*, and *CD98* (system l). System y+ is an important transporter of lysine and Arg in the placenta [51,52], which may explain the higher expression of *CAT1* in Arg placenta compared to Ctrl, which was in accordance with plasma Arg concentrations demonstrated by van der Linden et al. [18]. *SNAT1*, as part of system A, transports small non-essential AA, such as glycine, histidine, and methionine [53] and, similarly, *LAT2* and *CD98* (system l transporters) are obligatory exchangers of neutral AA, such as histidine and methionine [54]. Accordingly, van der Linden et al. [18] and McCoard et al. [20] identified concentrations of glycine, histidine, and methionine to be higher in plasma of Ctrl umbilical artery and vein, and fetal plasma, compared to Arg, suggesting that AA availability affected the expression of AA transporter genes.

Arg supplementation had no effect on any of the glucose transporters evaluated, which is consistent with the findings of McCoard et al. [20], where differences in plasma glucose concentrations between Arg and Ctrl fetuses were not evident. These results suggest that maternal Arg supplementation may not influence glucose transport in the placenta.

The growth factors *IGF2* and *IGF1* were initially investigated due to their sensitivity to changes in maternal nutrition and their influence on nutrient allocation, hormone secretion, and placental restructuring to promote fetal growth and development [55,56,57,58]. *IGF2,* but not *IGF1,* expression was found to be higher in Arg than Ctrl placentas. In mice, *IGF2* expression has been shown to affect placental growth, passive permeability, and nutrient transport [58,59,60], and Arg indirectly induces the expression of *IGF2* [60,61]. The higher expression of *IGF2* found in Arg compared to Ctrl in this study could, therefore, suggest greater nutrient transfer and permeability in the placenta in response to Arg supplementation of the dam. Furthermore, van der Linden et al. [18] found the weight of placentas and cotyledons to be heavier in Arg compared to Ctrl at birth, and Sibley et al. [59] demonstrated direct links between *IGF2* and placental weight in mice. *IGF2* may act similarly in the ovine placenta, which may explain the findings of van der Linden et al. [18]. *IGF1* expression in the placenta was not affected by supplementation in the present study, which is consistent with no difference in plasma *IGF1* in either the ewes or fetuses in the parent study [20].

Tissue-by-supplementation interactions were identified for the angiogenic factors *VEGFR2* and *FGFR2*. *VEGFR2* is one of two receptors for the vascular endothelial growth factor (VEGF), one of the major proteins that influences angiogenic activity in the placenta [49,62,63,64]. Utero–placental blood flow increases during pregnancy, and the placenta produces VEGF throughout gestation to promote angiogenesis and permeability [65,66]. *VEGFR2* expression was previously demonstrated to be higher in cotyledon than in caruncle tissue of twin-bearing ewes compared to single- and triplet-bearing ewes by van der Linden et al. [18]. Higher expression of *VEGFR2* in Arg cotyledons compared to Ctrl cotyledons could indicate greater angiogenic activity/vascularity initiated by Arg.

*FGFR2* is the receptor for basic fibroblast growth factor (FGF) and stimulates proliferation of both uterine arterial and fetal placental arterial endothelial cells and, thus, placental blood flow [2,3]. However, *FGFR2* was not affected by Arg supplementation in cotyledons, indicating different angiogenic mechanisms in Ctrl cotyledons. Both VEGF and *FGFR2* are stimulated by NO, and Arg stimulates NO production [2,3,63,67]. NO is an important regulator of blood flow by mediating vascular smooth muscle through the NO receptor *NOS3* and soluble guanylate cyclase (*GUCY1B1/GUCY1B3*). However, less than 2% of Arg is utilized for NO and polyamine synthesis, while most goes into the arginase pathway [16], which may explain why no effects were found on *NOS3* and *GUCY1B1/GUCY1B3*, which are involved in the synthesis of NO [68]. Nevertheless, the higher expression of *VEGFR2* in Arg cotyledons compared to Ctrl indicates increased vascular or angiogenic activity, which may increase placental nutrient transport.

Supplementation-by-subtype effects were evident for *SLC6A14* of the system B AA transporter system, and the isoform *CAT4* of the AA system y+ and the angiogenic/hypoxia factor, *HIF1a*. The results suggest that Arg supplementation influenced *SLC6A14* gene expression in type A placentomes and, thereby, may increase the transport capacity of neutral AAs [39]. *CAT4* is a transporter of cationic AAs, such as Arg in human placenta [69]; however, in this study, *CAT4* was not affected by Arg supplementation. It is important to note that mean mRNA counts for *SLC6A14* and *CAT4* in the placenta were below 20, suggesting very low levels of expression, which may have masked some supplementation effects. The importance of *SLC6A14* and *CAT4* in the ovine placenta is unknown [70], and further research is needed to validate the importance of the respective genes.

Hypoxia has, in previous studies, been suggested to be associated with the morphologic shift from type A to D of placentomes in sheep [11,40]. Therefore, *HIF1a* was investigated in this study, as it is stimulated by hypoxia. *HIF1a* triggers a coordinated response of angiogenesis and arteriogenesis by inducing the expression of certain angiogenic factors, such as VEGF and its receptors and nitric oxide synthase, which results in an increase of oxygen delivery, or a metabolic adaptation to the reduced availability of oxygen [71,72]. Twin pregnancies have a natural negative effect on the oxygenation status of the fetal–placental unit at day 140 of gestation compared to single-bearing pregnancies, despite the ewes’ nutritional status [72]. This study found that Ctrl animals had lower expression of *HIF1a* in type C placentomes compared to that of Arg-treated ewes, but no differences were observed in type A and B placentomes. The results of this study may suggest that the effect of maternal Arg supplementation on *HIF1a* expression could affect angiogenesis and arteriogenesis to increase oxygen delivery in type C placentomes and, thus, mitigate hypoxia. Further research is needed to investigate the effects of increased *HIF1a* to understand the physiological mechanisms mediating the Arg supplementation-by-subtype interaction.

## 5. Conclusions

In summary, this study was able to identify the presence and quantify the abundance of 31 genes in the ovine placenta. The glucose transporter gene *GLUT1* was identified as the most abundant gene of those evaluated in the placenta at day 140 of gestation. Clear differences in gene expression were identified between caruncle and cotyledon tissue, highlighting that future studies should consider distinguishing these tissue types to evaluate placental function. Furthermore, changes in the expression of some of the transporter genes (e.g., *SN1* and *SLC6A14*) between placentome morphologic subtypes suggests that the morphologic change may influence the nutrient transport capacity in the ovine placenta, with an increase in nutrient transport capacity in type B placentome types. Therefore, this study partly accepts the hypothesis that the morphologic shift from A to B and C subtypes increases transport capacity.

Previous studies have highlighted the effects of Arg supplementation on fetal growth and development. To the knowledge of the authors, this is the first study to investigate the effects of Arg supplementation in the ovine placentome and suggest links between AA availability and abundance of AA gene transporters in the placenta. This was demonstrated by higher *CAT1* expression in the placentas from Arg-supplemented ewes than from Ctrl ewes, an isoform of the predominant Arg transporter system y+, highlighting that increased transport of Arg occurred. Furthermore, the increased *IGF2* expression demonstrated in this study may be linked to the increased placental growth demonstrated by van der Linden et al. [18]. Arg supplementation also affected the expression of *VEGFR2* and *HIF1a*, suggesting increasing effects on angiogenesis and arteriogenesis in cotyledons through increased *VEGFR2* and mitigated hypoxic effects in type C placentomes. Arg supplementation may improve the nutrient transport capacity through increased placental transport of Arg and increased expression of growth, angiogenic, and hypoxic factors.

## Figures and Tables

**Table 1:** Number of ovine caruncles and cotyledons of each subtype from placentas supporting individual fetuses from Arg-supplemented or control (un-supplemented) dams used in this study (number of ewes in brackets).

Placentome Subtype	Arg	Control
A	18 (7)	21 (9)
B	17 (8)	19 (10)
C	16 (6)	17 (7)

**Table 2:** Details of genes analyzed in the placentomes, including their function, identification, accession, and name.

Function	Gene ID	Accession	Name
Glucose transporter	*SLC2A1 (GLUT1)*	XM_015091913.1	Glucose transporter 1; solute carrier family 2, member 1
Glucose transporter	*SLC2A3 (GLUT3)*	XM_012159621.2	Glucose transporter 3; solute carrier family 2, member 3
Glucose transporter	*SLC2A4 (GLUT4)*	XM_012185632.1	Glucose transporter 4; solute carrier family 2, member 4
Glucose transporter	*SLC2A8 (GLUT8)*	XM_012162546.2	Glucose transporter 8; solute carrier family 2, member 8
AA transporter	*SLC38A1 (SNAT1)*	XM_012170444.1	SNAT1 System A; solute carrier family 38, member 1
AA transporter	*SLC38A2 (SNAT2)*	XM_012170436.2	SNAT2 System A; solute carrier family 38, member 2
AA transporter	*SLC38A4 (SNAT4)*	XM_012155123.2	SNAT4 System A; solute carrier family 38, member 4
AA transporter	*SLC7A5 (LAT1)*	XM_012159575.2	LAT1 System L; Solute carrier family 7, member 5
AA transporter	*SLC7A8 (LAT2)*	XM_012129264.1	LAT2 System L; Solute carrier family 7, member 8
AA transporter	*SLC3A2/4F2hc (CD98)*	XM_012146229.2	Heavy chain transmembrane protein for systems L and y+L; solute carrier family 3, member 2
AA transporter	*SLC7A7 (y^+^LAT1)*	XM_012129339.2	y+LAT1 System y+L; solute carrier family 7, member 7
AA transporter	*SLC7A1 (CAT1)*	XM_012142866.2	System y+; solute carrier family 7, member 1
AA transporter	*SLC7A2 (CAT2)*	XM_012159185.2	System y+; solute carrier family 7, member 2
AA transporter	*SLC7A4 (CAT4)*	XM_012164278.2	System y+; solute carrier family 7, member 4
AA transporter	*SLC1A4 (ASCT1)*	XM_012125788.2	System ASC
AA transporter	*SLC38A3 (SN1)*	XM_012122049.1	SN1 System N; solute carrier family 38, member 3
AA transporter	*SLC7A9*	XM_015104066.1	B(0,+)AT System b; solute carrier family 7, member 9
AA transporter	*SLC6A14*	XM_004022406.3	bBo+ System B; solute carrier family 6, member 14
AA transporter	*SLC1A3 (EAAT1)*	XM_004017043.2	EAAT1 System X; solute carrier family 1, member 3
AA transporter	*SLC1A2 (EAAT2)*	XM_012096234.2	EAAT2 System X; solute carrier family 1, member 2
AA transporter	*SLC1A1 (EAAT3)*	XM_004004350.3	EAAT1 System X; solute carrier family 1, member 1
Angiogenic factor	*VEGFR-1 (FLT1)*	XM_015098156.1	VEGF receptor 1; FMs-related receptor tyrosine kinase 1
Angiogenic factor	*VEGFR-2 (KDR)*	NM_001278565.1	VEGF receptor 2; Kinase insert domain receptor
Angiogenic factor	*FGFR2*	XM_012103045.2	FGF2 receptor
Angiogenic factor	*TEK (Tie2)*	XM_012153095.2	Angiopoietin-1 receptor/TEK receptor tyrosine kinase
Angiogenic factor	*NOS3*	XM_015095442.1	Nitric oxide synthase 3
Angiogenic factor	*GUCY1B1*	XM_015101545.1	Soluble guanylate cyclase NO receptor enzyme
Hypoxic marker	*HIF1a*	XM_015097105.1	Hypoxia-inducible factor 1
Growth factor	*IGF-1*	NM_001009774.3	Insulin-like growth factor 1
Growth factor	*IGF-2*	NM_001009311.1	Insulin-like growth factor 2
Stress marker	*HSD11B2*	NM_001009460.1	11 beta-hydroxysteroid dehydrogenase type 2
Reference gene	*Cyclophilin A*	NM_001308578.1	Peptidylprolyl isomerase A
Reference gene	*GADPH*	NM_001190390.1	Glyceraldehyde 3-phosphate dehydrogenase
Reference gene	*RPL19*	XM_004012836.2	Ribosomal protein L19

## Data Availability

The data presented in this study are available upon request from the corresponding author. The data are not publicly available due to privacy restrictions.
